# Standardization and validation of a cytometric bead assay to assess antibodies to multiple *Plasmodium falciparum* recombinant antigens

**DOI:** 10.1186/1475-2875-11-427

**Published:** 2012-12-21

**Authors:** Bartholomew N Ondigo, Gregory S Park, Severin O Gose, Benjamin M Ho, Lyticia A Ochola, George O Ayodo, Ayub V Ofulla, Chandy C John

**Affiliations:** 1 Department of Biomedical Science and Technology, Maseno University, Maseno, Kenya; 2 Department of Pediatrics, University of Minnesota, Minneapolis, MN, USA; 3 Center for Global Health Research, Kenya Medical Research Institute, Kisumu, Kenya

**Keywords:** Multiplex, Malaria, Antibodies, ELISA

## Abstract

**Background:**

Multiplex cytometric bead assay (CBA) have a number of advantages over ELISA for antibody testing, but little information is available on standardization and validation of antibody CBA to multiple *Plasmodium falciparum* antigens. The present study was set to determine optimal parameters for multiplex testing of antibodies to *P. falciparum* antigens, and to compare results of multiplex CBA to ELISA.

**Methods:**

Antibodies to ten recombinant *P. falciparum* antigens were measured by CBA and ELISA in samples from 30 individuals from a malaria endemic area of Kenya and compared to known positive and negative control plasma samples. Optimal antigen amounts, monoplex *vs* multiplex testing, plasma dilution, optimal buffer, number of beads required were assessed for CBA testing, and results from CBA vs. ELISA testing were compared.

**Results:**

Optimal amounts for CBA antibody testing differed according to antigen. Results for monoplex CBA testing correlated strongly with multiplex testing for all antigens (*r* = 0.88-0.99, *P* values from <0.0001 - 0.004), and antibodies to variants of the same antigen were accurately distinguished within a multiplex reaction. Plasma dilutions of 1:100 or 1:200 were optimal for all antigens for CBA testing. Plasma diluted in a buffer containing 0.05% sodium azide, 0.5% polyvinylalcohol, and 0.8% polyvinylpyrrolidone had the lowest background activity. CBA median fluorescence intensity (MFI) values with 1,000 antigen-conjugated beads/well did not differ significantly from MFI with 5,000 beads/well. CBA and ELISA results correlated well for all antigens except apical membrane antigen-1 (AMA-1). CBA testing produced a greater range of values in samples from malaria endemic areas and less background reactivity for blank samples than ELISA.

**Conclusion:**

With optimization, CBA may be the preferred method of testing for antibodies to *P. falciparum* antigens, as CBA can test for antibodies to multiple recombinant antigens from a single plasma sample and produces a greater range of values in positive samples and lower background readings for blank samples than ELISA.

## Background

Most studies that have determined antibody responses to *Plasmodium falciparum* antigens and vaccine candidates in human plasma samples have used enzyme linked immunosorbent assay (ELISA) [1,2]. Recent advances in bead-based flow cytometry have made multiplex cytometric bead assay (CBA) antibody testing an attractive alternative to ELISA testing. The Luminex^100^ system can simultaneously quantitate up to 100 different proteins, peptides, DNA fragments or RNA fragments from a 5 μl sample in one well of a microtiter plate [3]. The multiplex assay is a bead format assay in which each bead set is internally color-coded with different ratio of red to infrared dyes, such that the Luminex^100^ can classify each bed set separately. The beads in multiplex assay anchor the antigens, as opposed to ELISA where the surfaces of the wells of microtiter plate anchor the antigen. The Luminex^100^ has two lasers; one laser beam excites the internal colored dyes for classification of the bead sets, while the other laser excites the reporter fluorochrome phycoerythrin (PE) [4,5]. Through classification of the bead set, various bead sets are distinguished, which correspond to up to 100 different analytes that the machine can quantitate, while the amount of analyte present in the plasma, serum or supernatant is quantified by excitation of the reporter fluorochrome [6].

Prior studies have reported use of the multiplex assay for antibody determination to *P. falciparum* antigens [7-11], and comparison studies of multiplex antibody measurements and traditional monoplex ELISA have shown a high correlation [4,11,12]. However, these studies have used a variety of different protocols and test antigens. There are, to date, limited published studies that provide information on assay optimization conditions or comparisons of antigen and plasma concentrations for multiplex testing. To provide standards for testing that will allow wider use of this technique by other researchers, optimal parameters for multiplex assay were determined and compared with results of ELISA. The assessed assay characteristics included: the optimal malaria antigen amount for CBA, optimal plasma dilutions for both CBA and ELISA assays, plasma buffer choice for CBA, comparison of MFI between single vs. multiplex CBA, optimization of the numbers of microspheres per reaction for CBA, testing of CBA readout with multiple variants of an antigen in a single test, and comparison of optimized CBA with ELISA.

## Methods

### Plasma samples

For validation and standardization of the multiplex assay of antibodies to *P. falciparum* antigens, a plasma pool made of 30 plasma samples from adults living in a Ugandan area of seasonal malaria transmission (positive control pool samples) [13] and seven plasma samples from North American individuals never exposed to malaria (negative control samples) was used. For comparison of antibody testing by CBA and ELISA to multiple *P. falciparum* antigens, samples from 30 individuals from a malaria endemic area of western Kenya were used [14]. Kenyan samples were obtained from both children and adults, to enable testing of a broad range of antibody values. Written informed consent was obtained from the study participants or, in the case of minors, from their parent or guardian. Ethical approval for the study was obtained from Kenya Medical Research Institute National Ethical Review Committee and the Institutional Review Boards of Makerere University and University of Minnesota.

### *Plasmodium falciparum* recombinant and peptide antigens

The ten recombinant *P. falciparum* antigens used for testing were apical membrane antigen-1 (AMA-1, full length ectodomain, 3D7 and FVO strains), erythrocyte-binding antigen (EBA-175, non-glycosylated region II), glutamate rich protein (GLURP, conserved non-repeat N-terminal region, amino acids 25–514, R0; and repeat C-terminal region, amino acids 705–1178, R2, 3D7 strain), merozoite surface protein-1 (MSP-1_19_, E-KNG variant; MSP-1_42_, 3D7, FUP and FVO strains), and merozoite surface protein-3 (MSP-3, C-terminus, FVO strain) were used for testing*.* Recombinant AMA-1 was expressed in *Escherichia coli* and provided by David Lanar, Walter Reed Army Institute for Research. Recombinant MSP-1_42_ and MSP-3 were expressed in *Escherichia coli*, and recombinant EBA-175 was expressed in *Pichia pastoris*, and provided by David Narum, National Institutes of Health. Recombinant GLURP was expressed in *Escherichia coli* and provided by Michael Theisen, Statens Seruminstitut, Copenhagen, Denmark. Recombinant MSP-1_19_ expressed in *Saccharomyces cerivisiae*, was provided by the Malaria Research and Reference Reagent Resource Center (Manassas, VA), and originally deposited there by David Kaslow. Blank samples consisted of the plasma diluent alone.

### Coupling of recombinant antigens to microspheres for the cytometric bead assay (CBA)

Microspheres were purchased from Luminex Corporation (Austin, TX). The bead stock was resuspended by gentle inversion for 1 min. An aliquot of 612, 500 beads was removed and centrifuged at 16, 000 g (Labnet Wood bridge, NJ) for 3 min. After discarding the supernatant, 100 μl of distilled water was added and centrifuged at 16, 000 g for 3 min. Beads were resuspended in 80 μl of activation buffer, 100 mM monobasic sodium phosphate (Sigma, S3139); pH 6.2, by vortexing (Scientific Industries Bohemia, NY) and sonication, 20 sec each. To activate the beads for cross-linking to proteins, 10 μl of 50 mg/mlN- hydroxysulfosuccinimide sodium salt (Sigma, 56485) was added to the beads and mixed by vortexing for 10 sec at moderate speed. Next, 10 μl of 50 mg/ml N-[3-dimethylaminopropyl] – N`-ethylcarbodiimidehydrochloride (Sigma, E1769) was added and the beads mixed again by vortexing for 10 sec at moderate speed. All incubations of beads were performed in the dark (covered with foil). The bead mixture was rotated on a rotary shaker (Labnet Edison, NJ) at room temperature for 20 min and vortexed for 10 sec at 10 min and at 20 min, both at moderate vortexing speed. Beads were pelleted by centrifuging at 16, 000 g for 5 min and washed twice with 250 μl of 100 mM morpholineethane sulfonic acid (MES), (Sigma, M2933), pH 6.0 buffer. Finally, beads were centrifuged at 16, 000 g for 5 min to pellet. To coat the beads with antigens, pelleted beads were resuspended with the relevant antigen and the volume adjusted to 500 μl per reaction by addition of coupling buffer (100 mM MES pH 6.0). Beads were conjugated to 0.5, 1, 2 and 5, 10 and 1,000 μg of different antigens. The antigen and activated beads mixture was incubated on a rotary shaker for 2 h at room temperature in the dark to allow bead coupling to occur. After being coated with proteins beads were centrifuged at 16, 000 g for 3 min and washed twice with 250 μl of PBS-TBN (PBS, 0.1% BSA, 0.02% Tween, 0.05% sodium azide) and resuspended in 200 μl of PBS-TBN. To determine the percentage recovery after the coupling procedure, coupled beads were counted on a haemocytometer (Hausser Scientific Horsham, PA).

### Testing for IgG antibodies to *P. falciparum* antigens by CBA

The volume of working solution (50 μl/ well) was calculated together with the number of beads that would result in 1,000 beads/region/well or 5,000 beads/region/well. Bead stocks were then combined in a 15 ml amber conical tube and diluted with PBNT (0.1% BSA, 0.05% Tween 20, 0.05% sodium azide in PBS) to result in 100 microspheres/μl or 20 microspheres/μl. 96 well-millipore microtiter plates (MABVN 1250, Millipore corporation, Billerica, MA) were pre-wetted with 100 μl of PBNT/well and aspirated using a millipore vacuum manifold and 50 μl of working bead solution was transferred to it. Plasma samples were thawed at room temperature, mixed and centrifuged at 16,000 g for 3 min. Plasma was diluted through a series of concentrations: 1:100, 1:200, 1:400, 1:1000, 1:2000 and 1:4000. Plasma samples were diluted in either buffer A (1xPBS, 0.1% BSA, 0.05% Tween 20, and 0.05% sodium azide) or buffer B (1xPBS, 1% BSA, 0.05% Tween 20, 0.05% sodium azide, 0.5% polyvinylalcohol, and 0.8% polyvinylpyrrolidone). Buffer A is the standard buffer used by other studies of multiplex CBA for *P. falciparum* antigens [9], while buffer B has been used for multiplex antibody testing to other antigens and found to decrease background reactivity [15].

Fifty μl of diluted plasma was added to each of the well of the microtiter plate. The plasma was mixed with the beads three times by pipetting up and down. The plates were incubated in the dark on a shaking microplate shaker (IKA®MTS, Wilmington, NC) at 600 rpm for 30 sec, followed by 300 rpm for 30 min. Plates were aspirated using a millipore vacuum manifold and washed twice with 100 μl/well of PBNT, and beads were resuspended in 50 μl PBNT by pipetting. 50 μl of diluted 1:1,000 goat antihuman IgG (gamma- chain specific, F(ab`)2 fragment-R-phycoerythrin (Sigma, P-8047 St. Louis, MO) in PBNT was added to each well, and incubated in the dark with shaking at 600 rpm for 30 sec, followed by 300 rpm for 30 min. Plates were aspirated using a millipore vacuum manifold and washed twice with 100 μl/well PBNT. The beads were resuspended in 100 μl PBNT by mixing and analysed on bioplex machine (Hercules, CA). The reader was set to read a minimum of 100 beads with of unique fluorescent signature/region and the results expressed as median fluorescence intensity (MFI).

### Testing for IgG antibodies to *P. falciparum* antigens by ELISA

To validate the multiplex assay, similar plasma samples were tested for IgG antibodies to the same *P. falciparum* antigens by enzyme-linked immunosorbent assay (ELISA). Recombinant antigens were dissolved in 0.01 M PBS to concentrations: 0.1 μg/ml for (AMA-1 3D7, AMA-1 FVO, EBA-175 and GLURP-R2), 0.2 μg/ml for (MSP-1_42_ FVO, MSP-1_42_ 3D7, and MSP-1_42_ FUP), 0.5 μg/ml for (GLURP-R0, MSP-1_19_ and MSP-3 FVO). These antigen concentrations were found to be optimal in previous studies for AMA-1, EBA-175, MSP-1_42_[1], GLURP-R0 [16-19], GLURP-R2 [16,18], MSP-1_19_[20] and MSP-3 [21]. Fifty microlitres of antigen solution was added to Immulon-4 plates (Dynex Technologies,Chantilly, VA). Following overnight incubation at 4°C, washing with PBS**-**0.05% Tween 20, and blocking in 5% (wt/vol) nonfat powdered milk in PBS, duplicate 50 μl samples of serum diluted through a series of concentrations ranging from 1:50, 1:100, 1:200, 1:500, 1:1000, and 1:2000 in 5% powdered milk were added to wells and incubated for 2 h at room temperature. After washing with PBS −0.05% Tween 20, 50 μl of alkaline phosphatase-conjugated goat anti-human IgG (Jackson ImmunoResearch, West Grove, PA) diluted 1:1,000 in 5% powdered milk was added and incubated for 1 h. After extensive washing with PBS-0.05% Tween 20, *p*-nitrophenylphosphate was added in accordance with the manufacturer’s instructions (Sigma, S0942 St. Louis, MO). The optical density (OD) was measured at 405 nm (Molecular Devices, Sunnyvale, CA).

### Statistical analysis

The degree of association between MFI or OD values was assessed using Pearson’s correlation (*r*). Comparison of MFI values with Buffer A *vs*. Buffer B was done using Student’s t-test. All statistical tests were 2-sided and a *P* value of less than or equal to 0.05 was considered to be statistically significant for all comparisons. Analyses were conducted with Stata software version 10.0 (Stata Corporation, College Station, TX).

## Results

### Optimal amount of antigen for multiplex CBA

To determine optimal amounts of antigen for testing, 612,500 beads were coupled with differing amounts of antigens (Table 1). For antigen amount testing, the coated beads were incubated with plasma diluted to 1:200 from the positive pool sample (a mixture of plasma from 30 adults from a malaria seasonal transmission area of Uganda). Antigen amounts of 0.5, 1, 2, 5 and 10 μg of antigen were each used to coat duplicate wells, and a positive pool sample was placed in each well. The optimal antigen amount was the amount that yielded the highest average MFI value. The optimal amount for MSP- 1_42_ FUP,MSP- 1_42_ 3D7 and GLURP-R0 was 0.5 μg; for AMA-1 3D7, MSP-1_42_ FVO,MSP-3 FVO was 1 μg; for AMA-1 FVO and EBA-175 was 2 μg; and for MSP-1_19_ was 10 μg. The optimal amount for GLURP-R2 was not tested separately, and the same amount as for GLURP-R0 (0.5 μg) was used in multiplex testing.

**Table 1:** Parameters tested in the CBA assay

**Experimental condition tested**	**Values tested**	**Type and number of plasma samples used**	**Outcome**
Amount of antigen	0.5, 1, 2, 5 and 10 μg	Positive pool (see Methods) in duplicate wells per antigen amount	Different antigens had different optimal amounts (see Results)
Plasma buffer component	BSA, polyvinyl alcohol, polyvinylpyrrolidone concentrations	North American (non-malaria exposed) control pool and positive plasma pool	Buffer B had similar MFI values to Buffer A for positive plasma pool samples, but lower MFI values than Buffer A for NA controls
BSA			
Polyvinylalcohol	Buffer A (0.1%, 0%, 0%) *vs*. Buffer B (1%, 0.5%, 0.8%)		
Polyvinylpyrrolidone			
Plasma dilution	1:100, 1:200, 1:400, 1:1,000, 1:2,000 and 1:4,000	30 samples from persons from a malaria endemic area, 7 North American control samples and duplicate positive pool plasma samples	Optimal 1:100 or 1:200
Assay format	Monoplex (each Ag)	8 malaria endemic plasma samples	Multiplex and monoplex gave similar values
	Multiplex (10 different Ags)		
Number of microspheres per reaction	5000 beads/analyte/well, 1000 beads/analyte/well	3 North American, 16 malaria endemic samples	1000 and 5000 beads/analyte/well gave similar values
Reproducibility	0 day	Positive pool, 2 North American and 3 malaria endemic samples	Highly reproducible results
	7 days later		

Parameters optimized in the multiplex cytometric bead based assay (CBA) assay with a set of variables investigated, description of samples used and standardized values that gave optimal results.

### Monoplex and multiplex CBA formats

**Figure 1:**
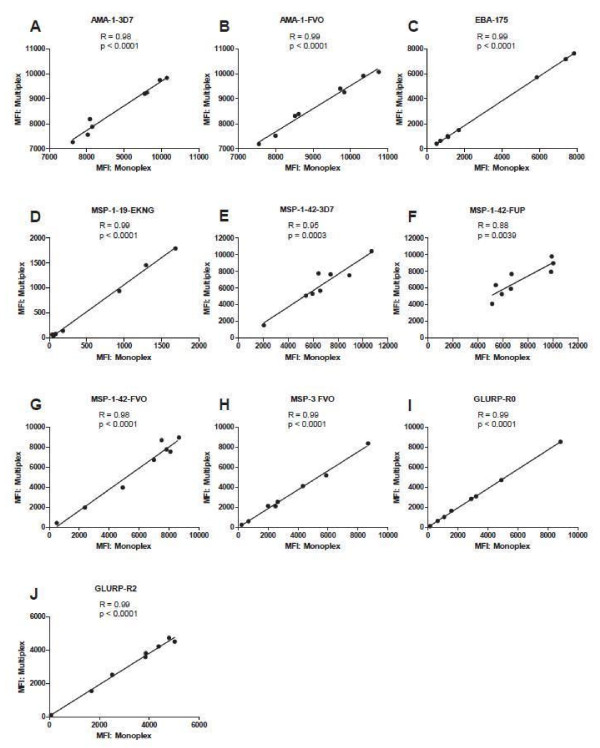
**Correlation of data between monoplex and 10-plex CBA in testing plasma samples by antigen.** Plasma from individuals in a malaria endemic area, (n = 8).

To determine how multiplexing influences MFI values in CBA assay, monoplex and multiplex assays were compared using the full 10-plex of *P. falciparum* antigens. Plasma IgG antibodies against each antigen were tested in a monoplex format (10 antigens tested separately) and as a 10-plex assay (in which antibodies to all antigens were tested in a single reaction well). For this experiment, eight plasma samples from individuals from malaria endemic area were used, and 5,000 beads/region/well were tested (Table 1). Plasma samples were diluted 1:200 (Figure 1). Results showed that MFI values for monoplex and multiplex were similar and statistically significant correlations between the two formats for all antigens was observed, with a Pearson’s correlation coefficient (*r*) ranging from 0.88 to 0.99, and all *P* values <0.0001 except for MSP-1_42_ FUP, *P* = 0.004 and MSP-1_42_ 3D7, *P* = 0.0003. Importantly, microspheres coupled to different allelic variants of the same antigen did not differ in monoplex *vs* multiplex testing, demonstrating that there were no significant problems with cross-reactivity in multiplex testing, even with multiple variants of the same antigen included in testing.

### Optimal plasma dilution for multiplex CBA and ELISA assays

**Figure 2:**
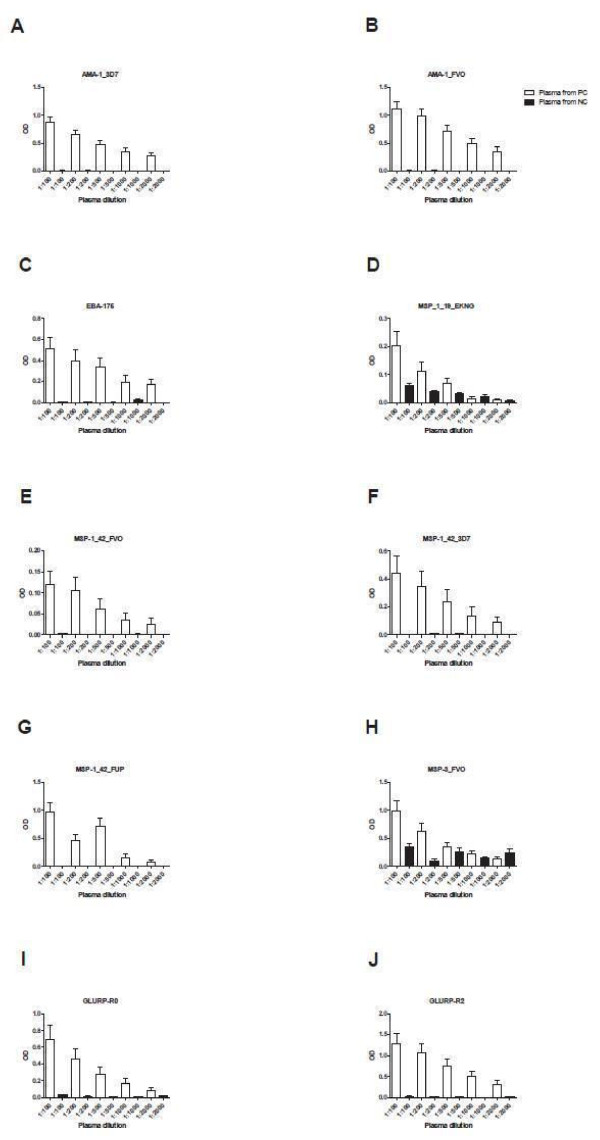
**Effect of plasma dilution on IgG antibody ELISA OD values of plasma from persons in a malaria endemic area, (n = 30) and persons never exposed to malaria, (n = 7).OD = optical density.** OD values on Y-axis differ according to antigen.

**Figure 3:**
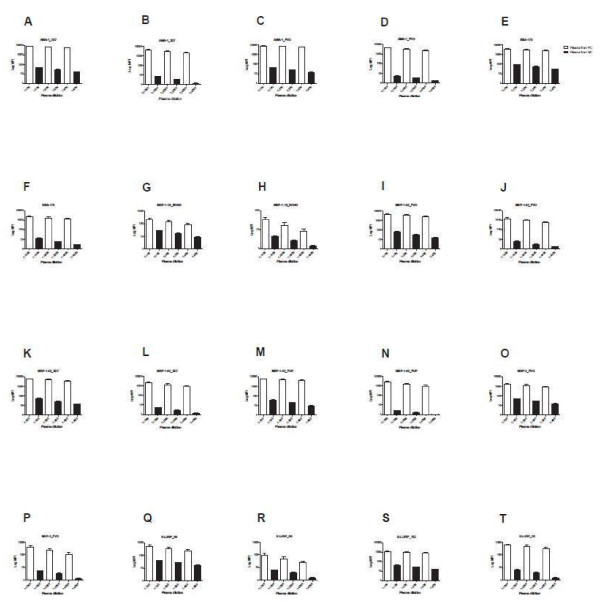
**Effect of plasma dilution on IgG antibody CBA logarithm MFI values of plasma from persons in a malaria endemic area, (n = 30) and persons never exposed to malaria, (n = 7).** MFI = Median Fluorescence Intensity.

**Figure 4:**
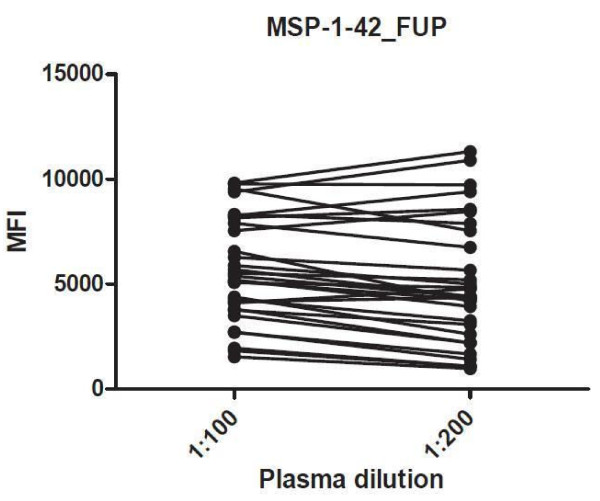
**MFI values for antibodies to MSP-1**_**42**_**FUP in individuals from a malaria endemic area, (n = 30) at 1:100 and 1:200 plasma dilutions.**

Optimal plasma dilution was determined by both multiplex CBA and ELISA for each antigen. Thirty samples from individuals from a malaria endemic area of Kenya, seven samples from North Americans never exposed to malaria and positive pool plasma samples were used for this testing (Table 1). Plasma was diluted to concentrations of 1:100, 1:200, 1:400, 1:1000, 1:2000 and 1:4000 for CBA, and 1:100, 1:200, 1:500, 1:1000 and 1:2000 for ELISA. Different concentration ranges were chosen because OD values of ELISA decreased more rapidly than MFI values, such that values at 1:2000 were extremely low and approached North American control values for instance MSP-3 and MSP-1_19_ (Figure 2). In contrast, the MFI values of CBA at 1:2000 were still well above North American control values. Multiplex CBA testing demonstrated consistently low MFI values for samples from North Americans never exposed to malaria, and these values generally decreased as plasma dilution increased (Figure 3). Plasma dilution of 1:100 provided the highest OD on ELISA testing for all antigens while mean MFI values were highest in malaria endemic samples diluted at 1:100 for all antigens. Nine samples showed a small increase in MFI from a 1:100 to a 1:200 dilution for the MSP-1_42_ alleles, suggesting a minor prozone effect, but the differences were smaller than the decreases seen with a decrease in dilution from 1:100 to 1:200 with all other samples. An example was for MSP-1_42_ FUP, for which antibody MFI values increased for five persons and decreased for 25 persons from a malaria endemic area (Figure 4).

**Figure 5:**
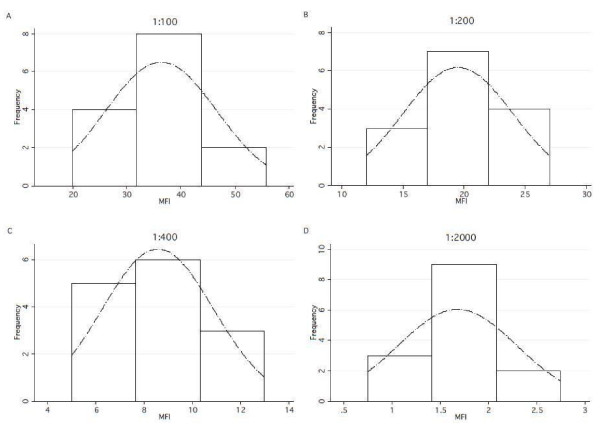
**Set of graphs across different dilutions overlaid with a normal density curve of CBA MFI valuesin plasma from persons never exposed to malaria to MSP-1**_**42**_**FUP, (n = 7).**

**Figure 6:**
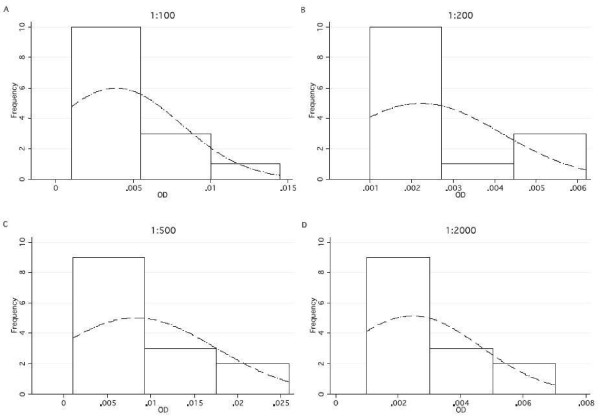
**Set of graphs across different dilutions overlaid with a normal density curve of ELISA OD values in plasma from persons never exposed to malaria to MSP-1**_**42**_**FUP, (n = 7).**

**Figure 7:**
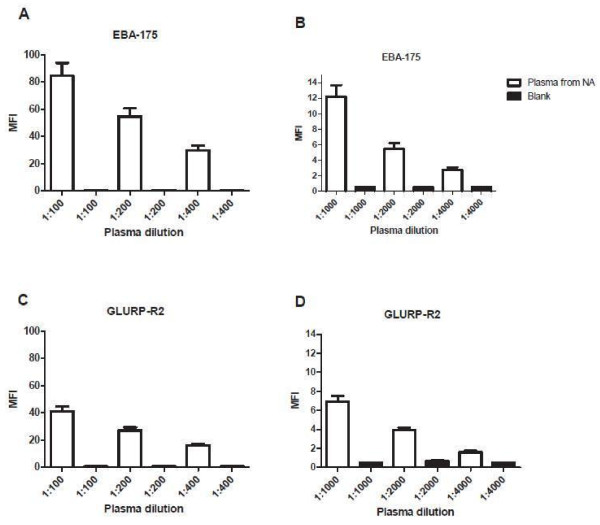
MFI values at different dilutions of plasma from persons never exposed to malaria, (n = 7) and blank to EBA-175 (7A and 7B) and GLURP-R2 (7C and 7D).

**Figure 8:**
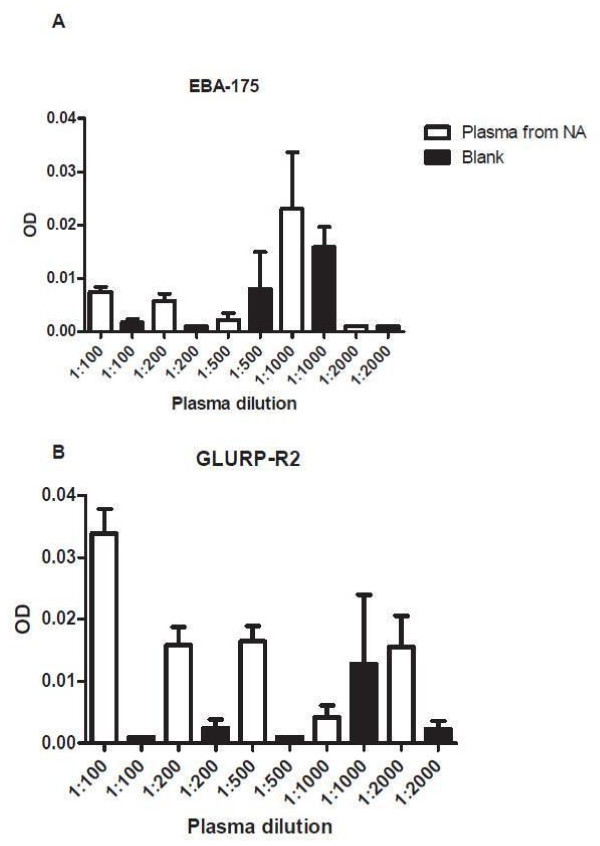
OD values at different dilutions of plasma from persons never exposed to malaria, (n = 7) and blank to EBA-175 (8A) and GLURP-R2 (8B).

Samples from North American individuals never exposed to malaria also showed an approximately normal distribution at all dilutions for 1:100, 1:200, 1:400 and 1:2000 dilutions for MSP-1_42_ FUP (Figure 5). In contrast, ELISA values for North American control samples showed much greater variability, did not decrease with increasing plasma dilution (Figure 2), and were often not normally distributed for 1:100, 1:200, 1:500 and 1:2000 dilutions (e.g., for MSP-1_42_FUP, Figure 6). MFI values of blank wells (beads in diluent buffer only) in CBA testing were almost uniformly 0 or 1, making background reactivity a non-issue with CBA testing (Figure 7), while blank wells in ELISA testing sometimes had low and sometimes high OD readings (Figure 8), creating more variability in assessment of the North American control and malaria endemic sample OD values, if blank OD values are subtracted from the control and malaria endemic sample values.

For CBA or ELISA testing in which there is no reference standard for antibody concentration, arbitrary units (AU) are often used to define antibody levels. Arbitrary units are used to standardize antibody values across plates [22]. For antibodies to *P. falciparum* antigens, AU is calculated using plasma samples from individuals (in this study, North Americans) never exposed to malaria. The mean OD or MFI of the North American plasma samples plus three standard deviations of the OD or MFI values is the cutoff value for 1 AU. The same North American samples are tested on every plate. Every test sample is then divided by the cutoff OD or MFI value to come up with an AU value, and a sample is categorized as “positive” by either multiplex or ELISA if the AU value us greater than ≥1. For this study, 30 North American control samples were tested, and seven samples chosen that are representative of the 30 samples, providing the same mean and standard deviation, for each plate, as testing all 30 samples on each plate would almost double the number of plates required. It is particularly important with this method, which has been used by others [22-26] that the samples provide a non-skewed range of MFI or OD values. The increased range, consistently normal distribution of North American control samples, and lower background values obtained with CBA as compared to ELISA, made calculation of arbitrary units more accurate and reliable with CBA than with ELISA.

For CBA, plasma dilutions of 1:100 or 1:200 generally provided the best combination of AU range and sufficient discrimination between North American control samples and blank (diluent alone) wells. At dilutions greater than 1:400, MFI values became so low (e.g., mean (SD) values for MSP-3 for 1:1000, 1:2000 and 1:5000 were 5.5 (1.4), 3.6 (0.9) and 1.4 (0.5), respectively) that although they were higher than the blank well values of 0 or 1, they were considered too close to the blank well values to be useful in calculating arbitrary units. Together, the findings lead to a conclusion that a plasma dilution of 1:100 or 1:200 was optimal for most antigens in the CBA assay to allow determination of antibody level for the antigen in an individual. Because of the few samples with increase MFI at 1:200 for the MSP-1_42_ antigens, a 1:200 dilution was chosen as the standard dilution for CBA.

AU values as calculated from ELISA testing had a much smaller range of minimum to maximum values than corresponding values calculated from CBA testing. For all antigens, a 1: 100 plasma dilution appeared to be the optimal dilution for ELISA testing.

### Reducing non specific background reactivity in CBA testing

To investigate reduction of non-specific background reactivity, two buffers were compared with plasma pooled from North American individuals (a mixture of seven North American plasma samples) and positive pooled samples. Pooled samples of each type were tested in quadruplicate for each buffer at a dilution of 1:200. Buffer A (1xPBS, 0.1% BSA, 0.05% Tween 20, and 0.05% sodium azide, pH 7.4) was compared to Buffer B (1xPBS, 1% BSA, 0.05% Tween 20, 0.05% sodium azide, 0.5% polyvinylalcohol, 0.8% polyvinylpyrrolidone, pH 7.4; see Methods section for rationale). The two buffers were compared using a six-plex CBA (AMA-1 3D7, AMA-1 FVO, EBA-175, MSP-1_42_ 3D7, MSP-1_42_ FUP and MSP-1_42_ FVO).

**Figure 9:**
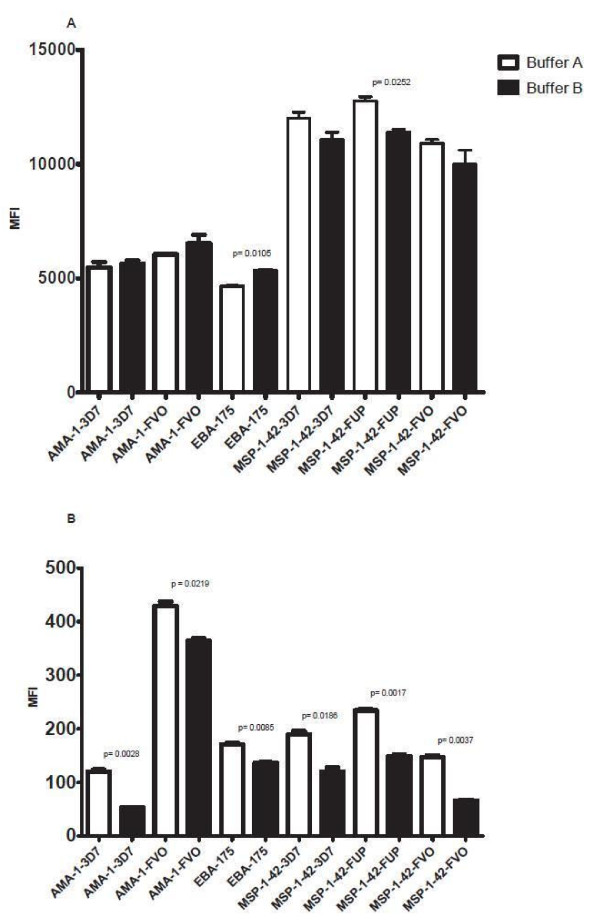
**Comparison of CBA MFI values to *P. falciparum *antigens using Buffer A *vs*. Buffer B. plasma pool from persons in a malaria endemic area (Figure 9A) and plasma pool from persons never exposed to malaria (Figure 9B).**

The two buffers yielded similar MFI values for the positive pool samples for all, (Figure 9A), with statistically significant differences only for EBA-175 (buffer B > buffer A) and MSP-1_42_ FUP (buffer A > buffer B). In contrast, MFI values for the non-malaria exposed North American plasma pool were significantly lower for buffer B for all six antigens (Figure 9B). The pooled positive plasma samples also had reduced bead aggregation when buffer B was used compared to buffer A. Buffer B thus provided greater differentiation in MFI values between plasma samples from malaria-exposed as compared to non-exposed individuals.

### Optimizing the number of beads per CBA analyte per well

**Figure 10:**
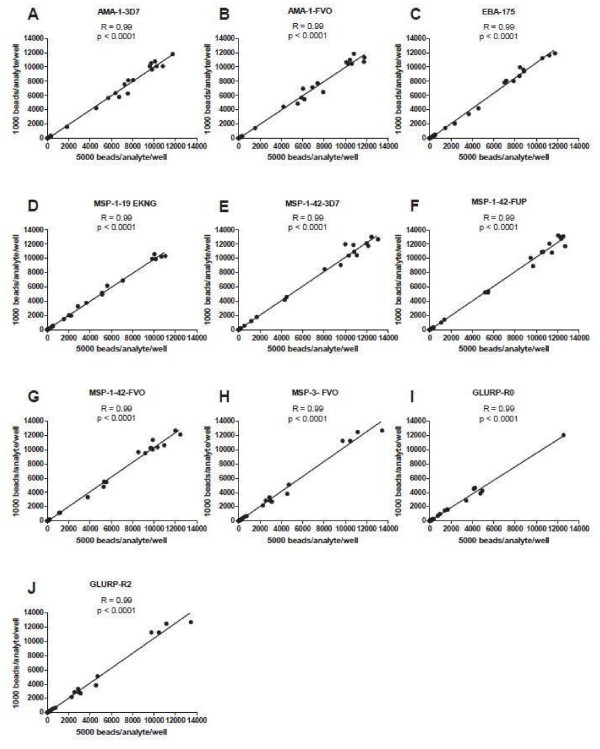
**Correlation of data from CBA testing with 5,000 beads/analyte/well *vs*. 1,000 beads/analyte/well.** Plasma from persons in a malaria endemic area (n = 16), plasma from persons never exposed to malaria (n = 3) and a positive plasma pool.

Standard testing protocols for CBA recommend 5,000 beads per analyte per well [3,11,12,27-29], but using large numbers of beads significantly increases expense. The number of beads per region per well used for each analyte throughout the optimization process was 5,000 as per microsphere manufacturer’s recommendations. To determine if similar results could be obtained with a lower number of beads, a comparison of MFI values obtained with 5,000 beads/analyte/well and 1,000 beads/analyte/well was performed. For this comparison purposes, sixteen plasma samples from individuals from a malaria endemic area of Kenya, three North American control samples, and one positive pool sample, all diluted to 1:200 were tested. MFI values were essentially identical for all antigens tested with the two different bead numbers (all *r*≥ 0.99, all *P* < 0.0001), (Figure 10). A minimum of 100 beads/well was read by the bioplex machine for each analyte.

### Reproducibility of the CBA coupling process

**Figure 11:**
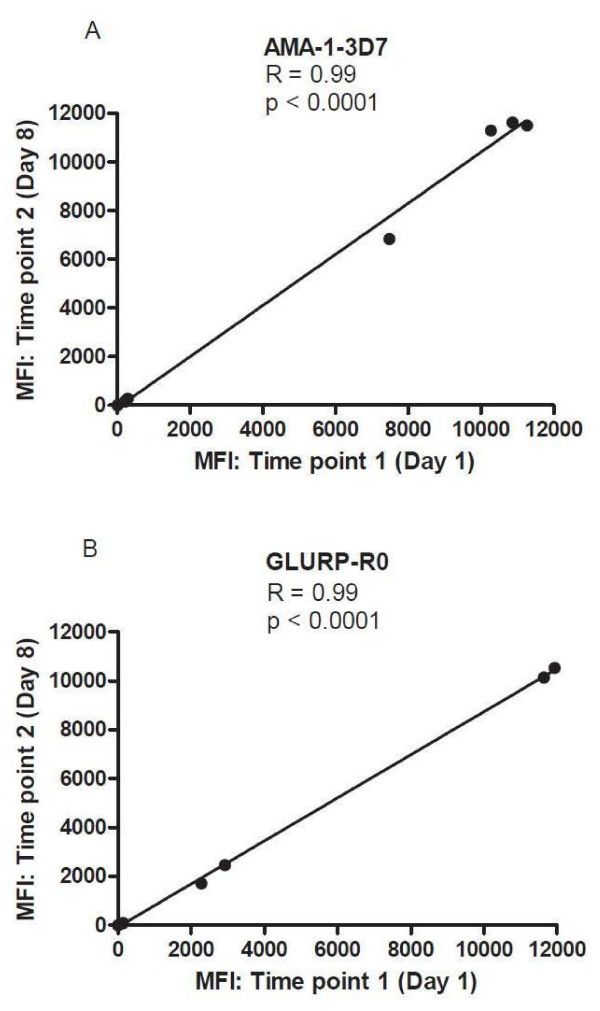
Regression plots of MFI data comparing 2-plex CBA results for IgG antibodies to AMA-1 3D7 and GLURP-R0 from plasma samples tested on separate days.

To evaluate the reproducibility of the coupling process, coupling reactions were performed on two different days (one week apart). Having established the optimal amounts of antigen suitable for coupling, 612,500 beads were coupled to respective optimal amounts to AMA- 1 3D7 and GLURP-R0 and 2-plex antibody detection was performed using a positive plasma pool sample, two North American control samples, and three samples from individuals from malaria endemic Kenya, all diluted 1:200 Absolute MFI values generated between the two coupling reactions were compared. The two coupling reactions produced almost identical results (both *r*≥ 0.99, *P* < 0.0001) (Figure 11).

### Concordance between multiplex CBA and ELISA assay

**Figure 12:**
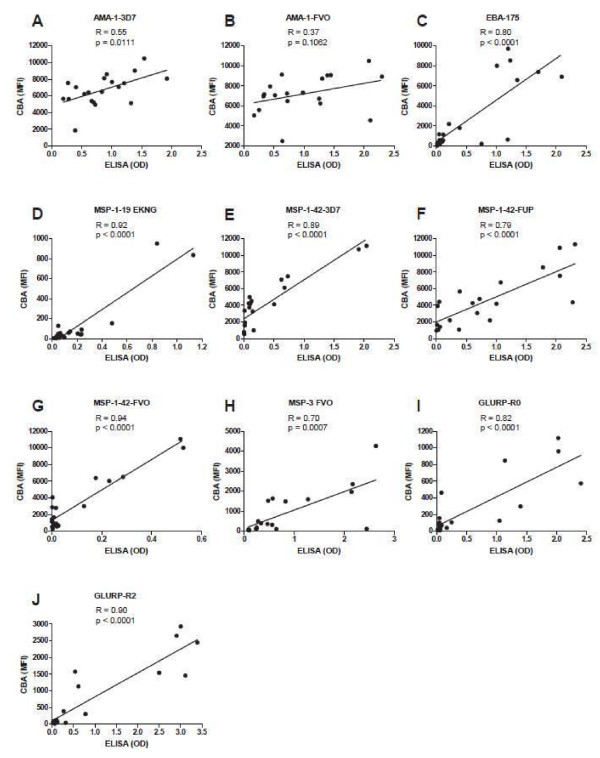
**Correlation of CBA MFI values and ELISA OD values for antibodies to *P. falciparum *antigens in individuals from a malaria endemic area, (n = 20).**

OD values by ELISA and MFI values by CBA were compared for samples from 20 individuals from malaria endemic Kenya and North Americans. Correlations between CBA and ELISA were strong (*r* > 0.7) and highly significant (*P* < 0.001) for all antigens except AMA-1, (Figure 12).

## Discussion

A number of recent papers have used multiplex CBA testing to assess for the presence of antibodies to *P. falciparum* antigens [8,10]. In this study, a comprehensive assessment of experimental parameters to optimize multiplex cytometric bead assay (CBA) testing of antibodies to *P. falciparum* antigens is provided. A recent paper by Ambrosino *et al.* characterized optimization of plasma dilution and amount of antigen for CBA malaria antibody testing and focused on testing antibodies to peptides of various *P. falciparum* antigens. This paper adds to the findings of prior papers by assessing optimal plasma dilution and antigen amount for several recombinant antigens not previously tested, investigating antigenic variants in the same multiplex testing, establishing optimal diluent buffer for the lowest background reactivity, and demonstrating that the testing can be performed accurately with significantly less beads than have previously been used in malaria studies. This study also demonstrates the specific advantages CBA may have over ELISA in antibody testing when there is no absolute reference standard, and relative units must be devised using samples from individuals without exposure to the pathogen to compare measurements of exposed populations.

Prior studies have documented similar results for CBA and ELISA for measurement of antibodies to *P. falciparum* or *Plasmodium vivax* antigens in malaria endemic populations [11,12]. Studies of antibodies to other infectious organisms have similarly shown that CBA compares well to established reference assays for these organisms [30-33]. Advantages noted over ELISA include specimen conservation, the ability to measure multiple analytes simultaneously, reduction in sample processing time due to kinetics of the assay, and cost minimization. The present study adds to this literature by demonstrating optimization of antigen concentration, plasma dilution, buffer solution, and number of beads. The study demonstrates that antibodies to antigenic variants can be tested in the same well (multiplex) and results are virtually identical to results of testing any variant alone by CBA (monoplex). It also provides evidence for the superiority of this testing as compared to ELISA testing in terms of providing less background, better differentiation of “positive” responses, and a broader dynamic range of antibody level values. Together, these findings support CBA as a precise, accurate and efficient alternative to ELISA for testing antibodies to multiple *P. falciparum* antigens, including multiple variants of the same antigen.

Determining appropriate working amounts of antigens is important in controlling surface density of the malaria antigens on the bead surface. Coupling small amounts may result in sub-optimal coating and low activity (less antibody binding), while too much antigen could result in precipitation and aggregation of the beads on the bottom of the microtiter well surface and this may impair the surface suspension antibody binding. Additionally, one would like to use the lowest amount of antigen that will provide the desired binding of antigens [34]. Reduction of background activity will enable malaria immunologists to accurately measure low-level antibodies, particularly in travellers [7,35,36] and in individuals under medication or individuals who are in recovery phase since antibodies have been shown to decline after malaria episodes [37,38].

Comparison between the 10-plex CBA and individual ELISA assays showed strong and highly significant correlations for all antigens except AMA-1 (Figure 12). The reasons for this discrepancy with AMA-1 are not clear. AMA-1 is among the most immunogenic *P. falciparum* antigens, and average MFI values for both AMA-1 variants were much higher than for other antigens, while OD values showed a similar distribution to that seen for other antigens. Low values were seen for both assays with North American controls, so it is not clear which assay more accurately reflected the range of AMA-1 antibody values in this population. This study documented an increase in MFI going from a plasma dilution of 1:100 to 1:200 for a few individuals for the antigen MSP-1_42_ only. This suggested evidence of a modest prozone phenomenon in these individuals. These findings demonstrate that some degree of compromise is required in decisions on a final dilution, as one dilution may not be the ideal for all antigens.

In the present study, long-term storage of multiplex beads, to see how long they could be used was not tested. Other studies have found that they provide slightly reduced MFI values after 7 and 12 months after initial bead coupling, if stored at 4°C [39,40]. Future studies will be required to confirm the study findings and assess longer storage periods of the coupled beads. Lyophilization of coupled bead sets, as used in a recent study [9], may provide an attractive alternative for long-term use of the same bead sets on different samples over time.

## Conclusion

The findings from the present study provide a basis for improved multiplex CBA testing, as the study testing provides information that will allow those testing by CBA to reduce background reactivity, optimize antigen concentration and plasma dilution, and use a five-fold lower number of beads for testing. Protocols will need to be individually tailored for specific antigens, but the results here provide a clear template for testing protocols that will allow for rapid customization and comparison testing. The present study also provides evidence of the increased dynamic range of CBA testing as compared to ELISA for this testing. The methods described in this study should assist in standardizing multiplex CBA antibody testing for multiple *P. falciparum* antigens, and allow it to become an important tool in seroepidemiology studies assessing population-based immunity in the face of changing transmission.

## Competing interest

The authors declare that no competing interests exist. The director of Kenya Medical Research Institute approved this article for publication.

## Authors’ contributions

BNO helped in the design aspects of the study, validated and performed assays, literature search, data analysis, drafting of the manuscript and transferring technology to field laboratory. SOG and BMH validated and performed assays and data acquisition. GSP assisted in data acquisition, helped in the design aspects of the study and intellectual content of the manuscript. GAA, LAO and AVO provided intellectual input and revision of the manuscript. CCJ conceived the idea and led the study design, implementation of the program and drafting of the manuscript and editing and review of the manuscript. All authors read and approved the final manuscript.
